# Biochar as an electron shuttle for reductive dechlorination of pentachlorophenol by *Geobacter sulfurreducens*

**DOI:** 10.1038/srep16221

**Published:** 2015-11-23

**Authors:** Linpeng Yu, Yong Yuan, Jia Tang, Yueqiang Wang, Shungui Zhou

**Affiliations:** 1 Guangzhou Institute of Geochemistry, Chinese Academy of Sciences, Guangzhou 510640, China; 2 Graduate University of Chinese Academy of Sciences, Beijing 100039, China; 3 Guangdong Institute of Eco-environmental and Soil Sciences, Guangzhou, 510640, China; 4 Guangdong Key Laboratory of Agricultural Environment Management, Guangzhou, 510640, China

## Abstract

The reductive dechlorination of pentachlorophenol (PCP) by *Geobacter sulfurreducens* in the presence of different biochars was investigated to understand how biochars affect the bioreduction of environmental contaminants. The results indicated that biochars significantly accelerate electron transfer from cells to PCP, thus enhancing reductive dechlorination. The promotion effects of biochar (as high as 24-fold) in this process depend on its electron exchange capacity (EEC) and electrical conductivity (EC). A kinetic model revealed that the surface redox-active moieties (RAMs) and EC of biochar (900 °C) contributed to 56% and 41% of the biodegradation rate, respectively. This work demonstrates that biochars are efficient electron mediators for the dechlorination of PCP and that both the EC and RAMs of biochars play important roles in the electron transfer process.

Biochar is a carbon-rich solid material produced by the thermal decomposition of diverse biomass species under oxygen-limited conditions^1^. Biochar has been widely proposed to be used in carbon sequestration, reduction of greenhouse gas emissions, and soil amendments in numerous agricultural and environmental applications^2^,^3^,^4^. The beneficial effects of biochar in these applications have mostly been attributed to its unique properties such as its highly porous structure, alkalinity and high ion-exchange capacity^5^,^6^,^7^. Because of its porous structure, biochar has also been reported to effectively immobilize a variety of environmental organic contaminants^8^,^9^,^10^,^11^, subsequently decreasing their bioavailability and ecotoxicity^12^,^13^. The sorption properties of biochar have been observed to vary dramatically under different pyrolysis conditions and feedstock sources^14^.

Organic contaminants sorbed onto biochars are generally assumed to be inactive for chemical and biological transformations. A series of recent studies, however, revealed that biochar can also catalyze the chemical transformation of contaminants^15^,^16^,^17^,^18^, a phenomenon that was previously confirmed for conventional black carbons such as activated carbon and graphite^19^,^20^,^21^,^22^,^23^,^24^. For example, Oh *et al.* reported that biochar can stimulate chemical reductive reactions of nitro herbicides^16^. A mechanical analysis indicated that biochar shuttled electrons between reductants (e.g., sulfides) and organic contaminants. The surface redox-active moieties (RAMs) of biochar were one factor potentially responsible for this mediation mechanism. Furthermore, Xu *et al.* recently indicated that biochar produced from red oak wood can catalyze hexahydro-1,3,5-trinitro-1,3,5-triazine (RDX) transformation by sulfides and that the chemical transformation rates of RDX are positively correlated to the electrical conductivity (EC, or termed π-π electron network associated with conductive graphite regions) of biochars^18^. The authors concluded that the EC of biochar, rather than its surface-oxygenated functional groups, accounts for its catalytic activity. In this case, the condensed aromatic structural regions in biochar could directly transfer electrons from the electron donors to the organic contaminants.

Similar to the role of biochar in chemical systems, biochar-mediated electron transfer in biological systems has also drawn considerable attention^25^,^26^. For instance, Kappler *et al.* reported that biochar could function as an electron shuttle to facilitate the electron transfer from *Shewanella oneidensis* MR-1 to Fe(III) minerals^27^. As a result, the rate and extent of microbial reduction of ferrihydrite were promoted. Recently, Tong *et al.* demonstrated that biochars amended to paddy soil can enhance the microbial transformation rate of pentachlorophenol (PCP)^28^. The enhancement was attributed to the increased extracellular electron transfer (EET) resulting from the biochar-promoted growth and metabolism of microorganisms in the soil. Although biochars have been reported to affect the electron transfer efficiency in various environments, as previously mentioned, little is known about how biochars shuttle electrons from microorganisms to the adsorbed contaminants or how the electrochemical properties of biochars affect the electron transfer pathway in microbial systems.

Thus, the objectives of this study were (1) to test the possibility that biochar stimulates the biodegradation of a widespread organic contaminant and (2) to identify the role of the EC (i.e., polycondensed aromatic structures) and surface redox-active groups of biochar in this process. Because its biodegradation pathway is well known, we selected PCP as a typical contaminant in this study. *Geobacter sulfurreducens*, which is capable of dechlorinating chlorinated contaminants under anaerobic conditions^29^,^30^, was used as the model microorganism. To address the effects of biochar surface redox-active groups and EC on the degradation rates of PCP, kinetic experiments in the presence of different biochars with various electron exchange capacities (EEC) and EC properties were conducted. Finally, a mathematic model was proposed to explain the mechanisms of biochar-mediated dechlorination.

## Results

### Physicochemical characteristics of biochars

**Figure 1:**
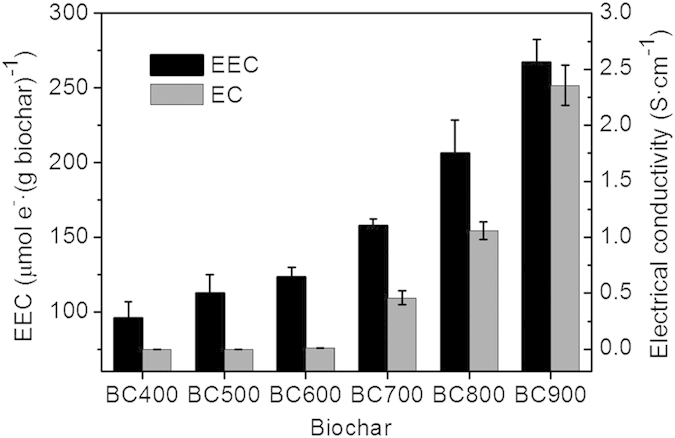
Changes in the electron exchange capacities (EECs) and electrical conductivities (ECs) of biochars as a function of the charring temperatures. EEC values of biochars were calculated from the sum of EACs and EDCs determined by MER and MEO, respectively. Error bars represent the standard deviation (SD) of triplicate measurements (n = 3).

The physicochemical properties of six different biochars prepared at a series of temperatures (400–900 °C) are shown in Table S1 (Supplementary Information). An elemental analysis indicated that the carbon contents of all of the biochars were comparable. The hydrogen content and H/C ratios of the biochars decreased gradually with increasing temperature, whereas the oxygen content and O/C ratios were not affected. The surface areas of the six biochar samples ranged from 5.46 m^2^·g^−1^ to 11.56 m^2^·g^−1^, with a small increase at relatively high charring temperatures. The ECs of biochars prepared at relatively low temperatures (400 °C to 600 °C) were low and changed slowly with the charring temperature; the conductivities then increased dramatically at high charring temperatures (>600 °C) (Fig. 1).

### Electrochemical properties of biochars

The redox properties of biochars were assessed through a mediated electrochemical analysis (Fig. S1a,b; Supplementary Information). As shown in Fig. 1, the EECs, which are representative of the abundance of RAMs in biochar particles^31^, increased nearly threefold with the increased charring temperature. FTIR spectroscopy was used to identify the contents of the quinone moieties on the surface of various biochars. As shown in Fig. S2a, the FTIR spectra indicated the presence of the C=O stretching of quinones (1645 cm^−1^)^32^. The intensity of the band revealed that varied contents of quinone moieties were present on the surface of the higher temperature treated biochars. However, the highest absorbance at this band was observed for BC400 with a relatively low EEC value, suggesting that besides quinone moieties, other redox-active moieties (e.g., phenolic moieties) might also contribute to the high EECs of biochars prepared at higher temperatures^31^. All of the biochars had comparable electron donating capacities (EDCs) (Table S1), and they were lower than their EACs. The low EDCs of biochars indicate that most of the RAMs of the tested biochars were in oxidized forms.

To further confirm the effect of the RAMs on the EEC values, biochars with altered quinone moieties were fabricated via chemical bonding, physical adsorption of quinone-enriched chemicals, and H_2_O_2_ pretreatment, as previously suggested^18^,^32^,^33^. The FTIR spectrum of MBC400 showed a small increase in the intensity of the band at 1645 cm^−1^ compared to that of BC400 (Fig. S2b). By contrast, the FTIR spectrum of MBC900 showed a significantly lower absorbance at 1645 cm^−1^ compared to that of BC900, demonstrating the elimination of select quinone groups by H_2_O_2_ pretreatment (Fig. S2c). CV measurements of MBC400 demonstrated the appearance of a new pair of redox-active peaks at approximately –0 V (vs. SHE). These peaks did not exist or were too weak to be observed in the CV curve of BC400 (Fig. S3). Notably, the ECs of MBC400 (3.23 μS·cm^−1^) and MBC900 (2.4 S·cm^−1^) were basically identical to those of the untreated BC400 and BC900, respectively. As a result of the treatments, the EEC value of MBC400 [143.4 ± 11.5 μmol e^−^·(g biochar)^−1^] and BC400-AQDS [110.2 ± 12.3 μmol e^−^·(g biochar)^−1^] increased, whereas that of MBC900 [181.7 ± 16.4 μmol e^−^·(g biochar)^−1^] decreased compared to their values before treatment.

### Biochar-stimulated PCP biodegradation

**Figure 2:**
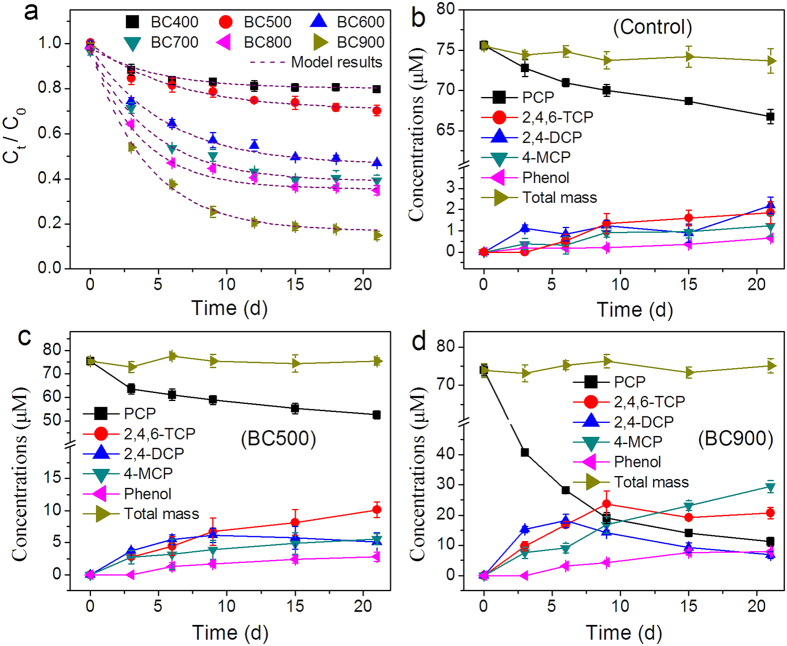
Biochar-mediated reductive dechlorination of pentachlorophenol (PCP) by *G. sulfurreducens* in the presence of different biochars (**a**), and kinetic changes of PCP degradation intermediate products in the absence of biochars (control, (**b**) or in the presence of BC500 (**c**) or BC900 (**d**). PCP (20 mg·L^−1^) was anaerobically incubated with *G. sulfurreducens* (an initial cell density of 0.9 × 10^10^ cells·L^−1^) in the presence of different biochars (2 g·L^−1^) at 30 °C. Error bars represent ± SD (n = 3).

The results from the anaerobic microbial degradation of PCP revealed that *G. sulfurreducens*, although defined as a model bacteria for extracellular respiration with a great current-generating ability, is a weak dechlorination-respiring microorganism that reduces PCP to less-chlorinated compounds (Fig. 2a). Only 11.1% (±1.2%) of the PCP was degraded by *G. sulfurreducens* after 21 d in the absence of biochars. However, this process was significantly accelerated in the presence of different biochars, yielding PCP degradation efficiencies of 20.3% (BC400), 29.7% (BC500), 53.1% (BC600), 60.7% (BC700), 65.1% (BC800) and 85.1% (BC900) under the identical conditions. The PCP biodegradation mediated by all biochars followed exponential decay equations over 21 d (Table S2), and the maximum PCP biodegradation rate (*k*_max_) increased from 0.65 mg·L^−1^·d^−1^ to 5.46 mg·L^−1^·d^−1^ as the biochar preparation temperature increased from 400 to 900 °C. These values were 2.9- to 24.8-times greater than that of the biochar-free control (0.22 mg·L^−1^·d^−1^). A GC/MS analysis of the PCP degradation intermediates after 21 d indicated the presence of 2,4,6-trichlorophenol (2,4,6-TCP), 2,4-dichlorophenol (2,4-DCP), 4-monochlorophenol (4-MCP) and phenol. These results indicated that the PCP degradation pathway involved reductive dechlorination reactions. The quantitative products of PCP were shown in Fig. 2b–d and Fig. S4. In the absence of biochars (the control), only a low level of PCP intermediates was detected during the 21-d incubation with *G. sulfurreducens*. However, the production of chlorophenol and phenol were significantly enhanced by BC500, with the highest catalyzing activities displayed for BC900. In the presence of BC900, the concentrations of 2,4,6-trichlorophenol and 2,4-dichlorophenol increased in the early period, but then decreased, suggesting that the PCP intermediates themselves could be degraded further by *G. sulfurreducens*. An analysis of total mass balance of PCP and its intermediates showed that almost all of the disappearing PCP was recovered in its dechlorination products (Fig. 2). Thus, the removal or extraction loss of PCP caused by other processes such as adsorption was negligible.

The adsorption behaviors of PCP by the different biochars were comparable (Fig. S5); no significant difference was observed in the equilibrium concentrations of PCP (2 ~ 3 mg·L^−1^) in the presence of different biochars after 48 h. No PCP degradation products in the cell-free biochar suspensions were detected by GC-MS (Fig. S4), suggesting that the chemical transformation of PCP by different biochars in the absence of cells was slow (Fig. S6). In other words, the dechlorination reactions mainly resulted from the biodegradation mediated by *G. sulfurreducens*. Changes in the biomass of *G. sulfurreducens* are shown in Fig. S7. In the absence of biochars, the quantity of *G. sulfurreducens* decreased to 86.7% of the initial biomass after 21 d. In the presence of BC400, BC700 or BC900, the biomass of *G. sulfurreducens* showed small fluctuations, but no great difference was observed (Fig. S7).

### The role of biochar EC and RAMs in PCP biodegradation

**Figure 3:**
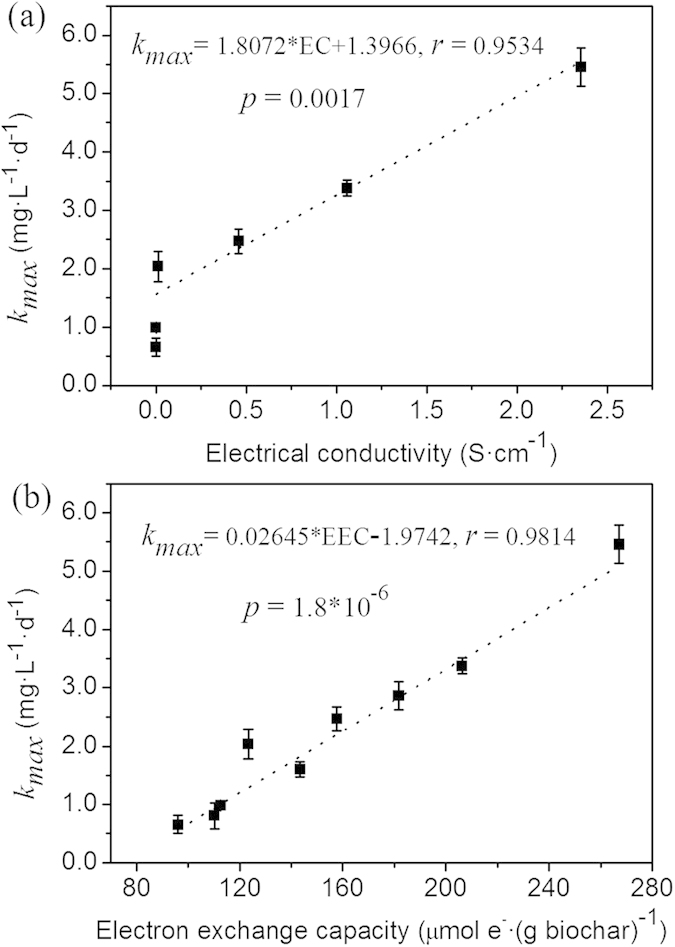
Correlations between (**a**) the maximum degradation rates (*k*_*max*_) and the ECs of biochars, and between (**b**) the *k*_*max*_ and the EECs of biochars. Dotted lines display the linearly fitted results. Error bars represent ± SD (n = 3).

**Figure 4:**
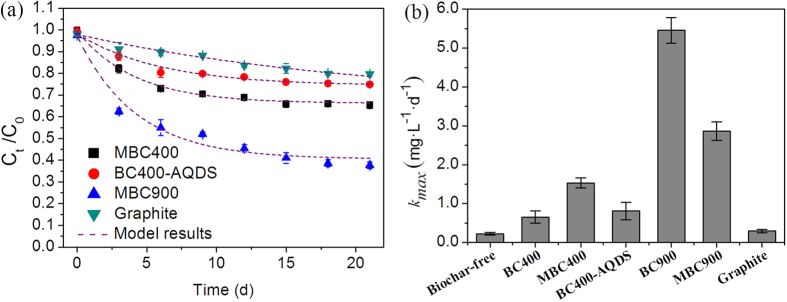
Kinetics of PCP biodegradation by *G. sulfurreducens* in the presence of modified biochars (**a**) and the comparison of the *k*_*max*_ (**b**) in the presence of various mediators. For these experiments, modified biochars were used to investigate the biodegradation kinetics of PCP under the identical conditions as mentioned in Fig. 2. Error bars represent ± SD (n = 3).

A strong linear correlation was observed between the ECs of the biochars and the *k*_max_ (*r* = 0.9534, *p* = 0.0017, Fig. 3a), and between the EECs of the biochars and the *k*_max_ (*r* = 0.9814, *p* = 1.8 × 10^−6^, Fig. 3b). However, distinguishing which of these two factors was primarily responsible for the accelerated dechlorination and degradation of PCP was difficult. Herein, the biodegradation behaviors of PCP in the presence of MBC400, BC400-AQDS, MBC900, and graphite particles were investigated to isolate the contribution of biochar RAMs from that of its EC. In the presence of BC400-AQDS and MBC400, the *k*_max_ of the PCP biodegradation (0.81 mg·L^−1^·d^−1^ and 1.53 mg·L^−1^·d^−1^, respectively) was enhanced by 23.3% and 134.0%, respectively, compared to that of BC400 (0.65 mg·L^−1^·d^−1^) (Fig. 4a). After 21 d, 25.2 ± 1.0% and 34.6 ± 1.5% of the PCP were degraded in the presence of BC400-AQDS and MBC400, respectively, compared to 22.1 ± 0.9% for BC400. The importance of the quinone groups was also demonstrated by MBC900 in the kinetic experiments of PCP biodegradation under the identical conditions. MBC900 exhibited a significantly decreased mediation activity in PCP biodegradation; its *k*_max_ (2.87 mg·L^−1^·d^−1^) was only 52.5% of that of the untreated BC900 (5.46 mg·L^−1^·d^−1^) (Fig. 4b). Accordingly, 63.3 ± 1.0% of the PCP in the reactors was biodegraded in the presence of MBC900 after 21 d, compared to 85.1 ± 1.9% for BC900. We also investigated the biodegradation of PCP in the presence of graphite powder; graphite powder is a conductive material with a low oxygen content (0.1% by weight). The *k*_max_ in the presence of graphite was 0.3-times greater than that of the biochar-free control, but was smaller than that of BC900. Although the EC of graphite powder (2.18 S·cm^−1^) was comparable to that of BC900, its redox mediation activity in PCP degradation was weaker, possibly explaining the differences in the *k*_max_ (Fig. 4b).

## Discussion

In this study, the different extents of biodegradation rate acceleration by the biochars primarily result from the differences in their physicochemical properties because the other conditions (biochar dosages, culture media and incubation conditions) were identical. Specifically, the increased (BC400-AQDS and MBC400) or decreased (MBC900 and graphite) mediation activity was only attributable to the prevalence of biochar RAMs, because the other properties such as the surface area and EC were not affected by the chemical treatments. The observed correlation between the biochar EEC and the *k*_max_ was consistent with the study of Yu *et al.*, which demonstrated that the rate constants for the biochar-mediated chemical reduction of nitrobenzene increased linearly with the amount of active oxygenated functional groups on the black carbon surface^17^. Phenolic moieties, quinone moieties and trace level of surface redox-active metals (such as Fe(III) and Mn) are possible RAMs responsible for the dechlorination reaction^31^. Several previous studies have demonstrated that immobilized humic substances on solid materials (alumina particles and anion exchange resins) can effectively mediate the reductive biotransformation of contaminants^34^,^35^,^36^. For example, Zhang *et al.* demonstrated that an insoluble Fe-humic acid complex could function as a solid-phase electron mediator for the microbial reductive dechlorination of PCP^37^. The enhanced degradation rates in this study can also be explained by the shuttling of electrons between cells and PCP by the adsorbed AQDS and modified hydroquinone on biochar particles.

The electron transfer from *G. sulfurreducens* to biochars may occur via a direct contact between the biochar and the conductive pili or outer membrane cytochromes^38^. Note that the PCP was also adsorbed by biochar particles. The coexistence of cells and PCP on biochar particles (Fig. S8) can facilitate the transfer of electrons between them. In this process, electrons generated by *G. sulfurreducens* are transferred through the π-π network of conductive graphite regions to reach the adsorption sites of PCP^39^. The conductive properties of biochars determine the electron transfer rate and, consequently, the reductive dechlorination rates of PCP. The role of the biochar EC was also similar to that of conductive magnetite nanoparticles previously reported by Aulenta *et al.*^40^, who demonstrated that magnetite nanoparticles served as an electrical conductor, accelerating the biodegradation of TCE. The electron transfer is typically energetically driven by the redox potential difference between *G. sulfurreducens* and PCP. The attached *G. sulfurreducens* has a rather negative potential (lower than −150 mV) that is determined by the outer membrane cytochromes of the cell^41^, whereas the standard redox potential of PCP (up to 2,3,4,5-tetrochlorophenol) is 0.399 V^32^. Therefore, the reduction of PCP by *G. sulfurreducens* is thermodynamically favorable on the surface of biochars.

Although the exponential decay equations successfully described the observed dynamic changes in the PCP biodegradation, they cannot elucidate how the physicochemical characteristics of biochars (RAMs and EC) affect the degradation rates. For the theoretical interpretation of the mediation mechanisms, a kinetic model that considers the relationship between the biodegradation rates and the electrochemical properties of biochars is needed. Table 1 shows the estimated parameters of the proposed model, and Fig. 2a shows the comparison between the fitted modeled data and the experimental data. The modeled data for biochars agree well with the observed kinetics of PCP biodegradation, with a correlation coefficient as high as 0.99 (Table 1).

The modeled degradation rate constant, *k*_*0*_, for direct reductive dechlorination was 3.31 d^−1^, agreeing with the value reported by Lin *et al.* for the kinetic model of PCP biodegradation by a pure culture of *Phanerochaete chrysosporium*^42^. The reducing rate of biochar surface RAMs by *G. sulfurreducens*, *μ*, was approximately 21% lower than that of AQDS^38^, which was attributed to the relatively low accessibility of these moieties in the solid phase compared to those in the aqueous phase. The estimated value of the parameter *R* for the biochar-free control, which represents the inhibition coefficient of PCP to cells, was comparable to the EC_50_ of 2,4-dichlorophenol to *G. sulfurreducens* reported by Duldhardt *et al.*^43^. Furthermore, the *R* values decreased with increasing EEC values and increasing ECs of biochars; these values were significantly lower than those of the biochar-free control. These results suggest that both the strong adsorption and accelerated degradation of PCP by biochars could alleviate the toxicity of PCP to *G. sulfurreducens*. By contrast, because of its low adsorption capacity, the *R* value for the graphite powder was approximately 2.9-times higher than that for BC400, significantly limiting its mediation ability. The estimated value of the saturation constant, *K*_*m*_, for the biochar-free control is on average 15-times greater than those in the presence of biochars. By contrast, the *K*_*m*_ for different biochars decreased gradually with *R*, implying that the biomass saturation on the biochar particles in terms of the biodegradation rate is more easily achieved with an increased biochar EAC. This biomass saturation concept is similar to the bio-reductive processes of Fe(III) oxides^38^. Such phenomenon likely results from a shift in the limiting step of PCP biodegradation from a biomass-determined mode to a process dominated by the chemical reaction rate between the reduced RAMs and the PCP when the biomass increases from values below *K*_*m*_ to values far exceeding *K*_*m*_^38^. The authors of previous studies have suggested that the value of *K*_*m*_ is dependent on the ratio of the particle size to the bacterial size^44^; however, different *K*_*m*_s for biochars with similar particle sizes (approximately 0.15 mm) indicated that other biochar properties (such as EECs and EC) can also influence the value of this parameter.

**Figure 5:**
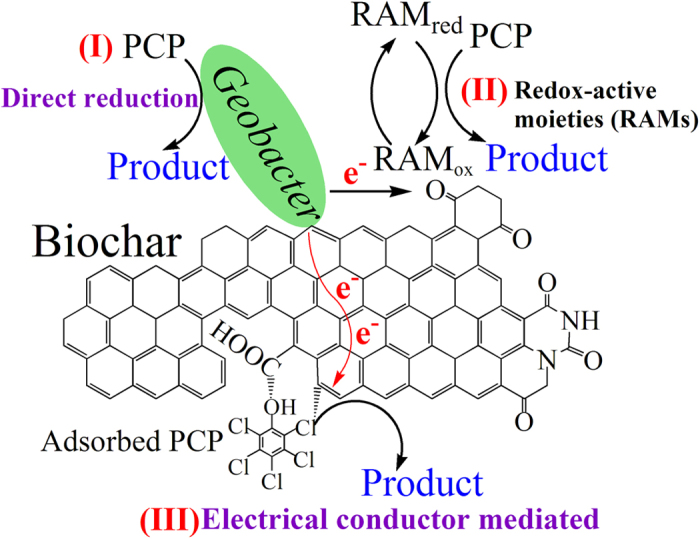
A schematic diagram of the direct and biochar-mediated electron transfer pathways in the process of PCP dechlorination by *G. sulfurreducens*.

The validity and applicability of the proposed model is verified by the good agreement between the modeled results and experimental data for both MBC900 and MBC400 (Fig. 4a). This agreement indicates that the model effectively describes biochar-mediated PCP degradation. The experimental and simulation results show that both the electrochemical properties (EEC) and EC of biochars display significant effects on the evolution of biochar reactivity during the PCP reduction reaction. Further analysis through the integration of the model equations shows that the stimulation of BC400 via the EC pathway was negligible but significantly increased with the increasing EC of biochar. In the case of BC900, the surface RAMs, its electrically conductive graphite regions, and the direct degradation pathway of PCP by *G. sulfurreducens* accounted for 56%, 41% and 3% of the degradation rate, respectively. This result demonstrated that three pathways for PCP degradation coexist in the presence of biochars, including the electron transfer from *G. sulfurreducens* to PCP via direct reduction, the mediation of biochar surface RAMs, and its electrically conductive graphite regions (Fig. 5). The rates of electron transfer in these pathways may be associated with the diffusive rate of the PCP in the medium, the regeneration of the reduced RAMs, and the relative abundance of graphite regions in the biochars.

In conclusion, we demonstrate that *G. sulfurreducens,* a weak dechlorination-respiration bacterium, can effectively reduce PCP in the presence of biochars. Both the biochar surface redox-active moieties and conductive graphite regions play crucial roles in the electron transfer process of the microbial dechlorination. Considering the ubiquity of black carbon and conductive minerals in the environment, these processes likely occur naturally at many contamination sites. This study highlights the varied mediating activities of biochars in the bioreduction of contaminants depending on their electrochemical properties.

## Methods

### Biochar preparation and characterization

Biochar was produced from rice straw obtained from a local paddy field in Guangdong Province, China. After being cut into 2–3 cm pieces, the rice straw pieces were placed into a cylindrical quartz tube in an electric furnace and pyrolyzed under a N_2_ flow of 1.2 L·min^−1^. The temperature of the furnace was programmed to increase at a rate of approximately 20 °C·min^−1^ and was held at different values (400–900 °C) for 1 h. After cooling to room temperature, the charred materials were milled to approximately 0.15 mm and sieved through a 100-mesh sifter. According to the charring temperature, the obtained biochars were designated as BC400, BC500, BC600, BC700, BC800 and BC900. The biochar elemental composition and surface area were determined using an elemental analyzer (Vario EL Cube, Elementar Co., Germany) and a Quantachrome QuadraWin (ASIQMO002-2, Contador Instrument Co., USA), respectively.

### Measurement of the electrical conductivity of biochar

The electrical conductivity (EC) of biochar was measured using the two-probe bed technique proposed by Mochidzuki *et al.* and by Xu *et al.* with a slight modification^18^,^45^. Specifically, the two-probe bed consisted of a pressure device, a hand-held milliohm meter and a cylindrical iron bed with two stainless steel pistons at the top and the bottom. The inner surface of the bed was completely insulated from the biochar and pistons by an embedded polypropylene material. After approximately 1.0 g of biochar sample was loaded into the bed, a high pressure (4 MPa) was applied, and the resistance of the whole bed was recorded.

### Measurement of the biochar electron-accepting capacity and electron-donating capacity

Electrochemical experiments were performed using an electrochemical workstation (CHI660D, Chenhua Co., Ltd., Shanghai, China) with a conventional three-electrode cell at ambient temperatures^46^. A graphite plate (2 × 2 cm), a platinum sheet and a saturated calomel electrode (SCE) were used as the working electrode, counter electrode and reference electrode, respectively. Chronoamperometry (CA) measurements were performed in a phosphate buffer solution (PBS, 0.1 M, pH = 7.0) with 0.1 M KCl electrolyte under constant stirring and N_2_ flow. To quantify the electron-accepting capacity (EAC) and electron-donating capacity (EDC) of biochar, the mediated electrochemical reduction (MER) and oxidation (MEO) were conducted at an applied potential of −0.49 V and +0.61 V (vs. SHE), respectively. A synthesized compound (zwitterionic viologen 4,4′-bipyridinium-1,1′-bis(2-ethylsulfonate)) and 2,2′-azino-bis(3-ethylbenzothiazoline-6-sulfonic acid) diammonium salt (ABTS, >98%, purchased from Sigma-Aldrich) were used as an electron shuttle for MER and MEO, respectively^31^,^47^. The total electron exchange capacities (EECs) of biochars, representing the entire capacity to donate and accept electrons, were obtained from the sum of the EACs and EDCs^31^.

### Biochar surface quinone modification

BC400 that were chemically modified by hydroquinone or adsorbed by anthraquinone-2,6-disulfonate (AQDS) were prepared for enrichment with quinone functional groups^33^,^18^. Additionally, surface quinone groups of BC900 were destroyed according to the method reported previously by Zhang *et al.*^32^, which is associated with the oxidization of the organic fractions of biochars. The modified biochar products were designated as MBC400, BC400-AQDS and MBC900 (see the detailed preparation procedures in the supplementary information).

### Microbial PCP degradation experiments

To prepare the cells for PCP biodegradation kinetic experiments, *Geobacter sulfurreducens* (PCA) was routinely cultured anaerobically in nutrient broth (NB) medium with acetate and fumarate (NBAF) at 30 °C as previously described^48^. Cells in the exponential growth phase were harvested by centrifugation.

Kinetic experiments of PCP biodegradation were conducted in 100 mL serum bottles containing 50 mL of fumarate-free NB medium with 15 mM acetate and different biochars (2 g·L^−1^) as the electron donor and electron shuttle, respectively. All media were buffered by bicarbonate solutions (pH = 7.0) and autoclaved at 121 °C for 20 min prior to use. PCP stock solution was sterilized by filtration at 0.22 μm and spiked into the media to give a final concentration of 20 mg·L^−1^. After the media was purged with N_2_/CO_2_ (80%: 20%) for 30 min, *G. sulfurreducens* were inoculated into the media (approximately 0.9 × 10^10^ cells·L^−1^) and the reactors were tightly sealed with rubber stoppers and aluminum caps. Each experiment was performed in triplicate under identical conditions in an incubator at 30 °C. The chemical transformation and adsorption of PCP by biochars were conducted using the identical procedure as above with the exception that no cells were added to the sterilized serum bottles. At a selected time interval, the serum bottles were vibrated vigorously for 30 min and sampled with a sterile syringe. The total content of PCP and its intermediate products was extracted and quantified by high-pressure liquid chromatography (HPLC) and gas chromatography-mass spectrometry (GC/MS), respectively, as previously described (see the details in the supplementary information)^28^. The microbial biomass of *G. sulfurreducens* was determined by real-time fluorescent quantitative PCR according to previous methods^28^,^49^. For the adsorption experiments, biochars were removed by filtration (0.22 μm) without the extraction process, and the PCP in the aqueous phase were determined by HPLC.

### Mathematical kinetic modeling of PCP biodegradation

Based on the kinetics results, the roles of biochar surface redox-active moieties (RAMs) and EC were considered in a mathematical model to explain the possible mechanisms of biochar-mediated PCP biodegradation. The model was analogous to those reported by MacDonald *et al.* and Pat-Espadas *et al.*^38^,^50^, who suggested a kinetic model for AQDS-mediated microbial reduction of ferrihydrite-coated sand and palladium(II), respectively. The present model employs the following assumptions: (1) the reduction rate of RAMs of the biochar was proportional to their concentrations and biomass; (2) the growth of *G. sulfurreducens* in all experiments was not considered, but the inhibition or decay of PCP on cells was considered; (3) *G. sulfurreducens* could reduce PCP in three ways: direct reductive dechlorination, RAMs-mediated reductive dechlorination, and conductive graphitic region-mediated reduction; and (4) the rate of graphitic region-mediated reduction of PCP was proportional to the biomass and biochar EC and to the concentration of PCP.

In the absence of biochars, PCP was dechlorinated by *G. sulfurreducens* via the direct contact pathway and the decay kinetics can be expressed as follows:


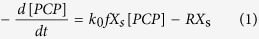



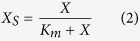


where *k*_*0*_ is the degradation rate constant, [PCP] is the remaining concentration of PCP in the media, *f* is the proportions of PCP in the aqueous phase, and *R* is a parameter reflecting the inhibition or decay on the cell microbial activity caused by PCP exposure. *X*_*s*_ represents the saturating function of biomass (*X*), and *K*_*m*_ is a saturation constant^38^,^50^.

When biochar or graphite was present in the systems, the second pathway of PCP dechlorination was a graphite region-mediated reaction. For this pathway, the degradation rate can be described as follows:


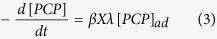


Here, *λ* is the electrical conductivities of the mediators, *β* is the reaction rate constant between the conductive graphitic region and PCP, and [PCP]_*ad*_ is the concentrations of PCP adsorbed by biochars.

For the RAMs-mediated reductive reaction pathway, the kinetic differential equations can be expressed as follows:


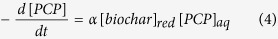



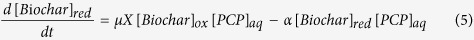



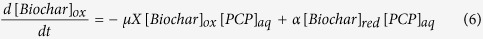



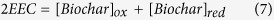


where *α* is the reaction rate constant between the reduced RAMs and the PCP, and [PCP]_*aq*_ is the concentrations of PCP in the aqueous medium. Eq. 5 and 6 represent the kinetic change of RAMs, where [Biochar]_red_ and [Biochar]_ox_ are the concentrations of the reduced and oxidized forms of RAMs, respectively, and *μ* is the rate constant for the microbial reduction of RAMs.

The proposed model equations for the kinetics of PCP biodegradation were coded as differential equations in Matlab 7.0 and solved using its ode45 program, which integrates the differential equations through a variable-step-size Runge-Kutta method. The Matlab optimization function ‘lsqcurvefit’ was used for model parameter estimation to minimize the differences between the modeled values and the real data^50^.

## Additional Information

**How to cite this article**: Yu, L. *et al.* Biochar as an electron shuttle for reductive dechlorination of pentachlorophenol by *Geobacter sulfurreducens*. *Sci. Rep.* **5**, 16221; doi: 10.1038/srep16221 (2015).

## Supplementary Material

Supplementary Information

## Figures and Tables

**Table 1:** Model parameter estimation of PCP biodegradation mediated by biochars (or graphite) in the presence of cells or only *G. sulfurreducens* (control).

Parameter	Control	Graphite	BC400	BC500	BC600	BC700	BC800	BC900
*R* (mg/(L·cell·d))	58.26	35.92	9.28	9.15	5.50	4.63	4.24	2.22
*f*	0.999	0.945	0.136	0.147	0.114	0.106	0.117	0.108
*K*_*m*_(cells/L)	3.18 × 10^11^	2.70 × 10^11^	2.96 × 10^10^	2.71 × 10^10^	2.34 × 10^10^	1.87 × 10^10^	1.72 × 10^10^	1.64 × 10^10^
*r*	0.9906	0.9740	0.9894	0.9743	0.9953	0.9966	0.9861	0.9982
*p*	1.2 × 10^−7^	1.3 × 10^−6^	4.9 × 10^−8^	1.4 × 10^−7^	1.5 × 10^−6^	4.5 × 10^−6^	1.1 × 10^−4^	2.6 × 10^−5^

*k*_*0*_ = 3.31 d^−1^; *μ* = 2.08 × 10^−10^  L·cell^−1^·d^−1^; *X* = 0.9 × 10^10^ cell·L^−1^.

*α* = 5.13 × 10^−2^ L·mol^−1^·d^−1^; *β* = 6.75 × 10^−14^ L·Ω·m·d^−1^.
