# Supplementary material for: Biochar as an electron shuttle for reductive dechlorination of pentachlorophenol by Geobacter sulfurreducens
Source: Sci Rep. 2015 Nov 23;5:16221. doi: 10.1038/srep16221 (PMC4655402; doi:10.1038/srep16221)
Supplement: Supplementary Information [file srep16221-s1.pdf]

Supplementary information

Biochar as an electron shuttle for reductive dechlorination of pentachlorophenol by *Geobacter sulfurreducens*

Linpeng Yu[1,2,3,*], Yong Yuan[3,4,*], Jia Tang[3,4], Yueqiang Wang[3,4] & Shungui Zhou[3,4]

[1]Guangzhou Institute of Geochemistry, Chinese Academy of Sciences, Guangzhou 510640, China

[2]Graduate University of Chinese Academy of Sciences, Beijing 100039, China

[3]Guangdong Institute of Eco-environmental and Soil Sciences, Guangzhou, 510640, China

[4]Guangdong Key Laboratory of Agricultural Environment Management, Guangzhou, 510640, China

Corresponding author: Shungui Zhou

Email: sgzhou@soil.gd.cn (S. Zhou)

Tel: +86-20-87025872, Fax: +86-20-87025872

*These authors contributed equally to this work.

Linpeng Yu & Yong Yuan

- 15 Pages
- 2 Table
- 8 Figures

List of Contents

Table S1. Physicochemical characteristics of biochars used in the present study.

Table S2. The fitted parameters of PCP biodegradation in the presence of different biochars.

Fig. S1 Mediated electrochemical reduction (a, MER) and oxidization (b, MEO) of different biochars by the graphite electrode at applied potentials.

Fig. S2 Fourier Transform Infrared Spectrum of (a) six different biochars, (b) BC400 and BC400 modified by hydroquinone (MBC400), (c) BC900 and H_2O_2-treated BC900 (MBC900).

Fig. S3 Cyclic voltammetry (CV) characteristics of BC400 and BC400 modified by hydroquinone (MBC400) in the phosphate buffer solution (0.1M, 0.1M KCl, pH=7.0).

Fig. S4 GC/MS chromatographs of PCP and its degradation products in the presence of different biochars.

Fig. S5 Adsorption of PCP by different biochars.

Fig. S6 Chemical transformation of PCP by different biochars in the cell-free NBAF culture mediums.

Fig. S7 Time courses of the biomass of *Geobacter sulfurreducens* during PCP dechlorination in the absence or presence of biochars.

Fig. S8 SEM images of *Geobacter sulfurreducens* growing on the surface of biochars during the reductive dechlorination of PCP.

Table S1. Physicochemical characteristics of biochars used in the present study

Samples	Elemental analysis						Electrical conductivity (S/cm)	Surface area (m^2/g)	EAC[a] μmol e^-/g biocar	EDC[a] μmol e^-/g biocar
	C	H	O	N	O/C	H/C				
	Percentage by weight (%)				Atomic ratio					
BC400	51.56	2.49	26.40	0.79	0.38	0.58	3.38E-06	5.46	76.9±6.9	19.3±3.7
BC500	53.06	1.74	23.57	0.68	0.33	0.39	1.74E-05	5.03	85.4±9.4	27.2±2.8
BC600	53.10	1.39	24.34	0.62	0.34	0.31	0.011329	5.41	112.1±4.9	11.2±2.0
BC700	52.51	1.26	23.24	0.58	0.33	0.29	0.460	8.95	145.2±3.4	12.5±1.3
BC800	48.32	1.21	25.83	0.79	0.40	0.30	1.06	11.56	194.0±17.8	12.3±4.4
BC900	46.90	1.18	24.96	0.78	0.39	0.30	2.36	10.85	258.3±14.7	8.9±0.6

[a]The results of biochar EACs and EDCs were expressed as mean ± SD (n=3).

Calculation of the maximum PCP degradation rate (k_{max}). The exponential decay equation was used to compare the biodegradation of PCP in the presence of different biochars and can be expressed as follows:

$$C_t = A_0 \cdot \exp(-\frac{t}{D_0}) + B_0 \qquad \text{Equation S1}$$

Where C_t is the concentration of PCP at different times, A_0 is the decay intensity constant, D_0 is the decay index, and B_0 is a constant. The fitted parameters are summarized in Table S2. The differential form of the equation (1) can be calculated as follows:

$$\frac{dC_t}{dt} = -\frac{A_0}{D_0} \cdot \exp(-\frac{t}{D_0}) = -k_{max} \cdot \exp(-\frac{t}{D_0}) \qquad \text{Equation S2}$$

Here k_{max} is obtained from the values of $\frac{A_0}{D_0}$, representing the observed maximum PCP degradation rate ($\frac{dC_t}{dt} \big|_{t=0}$).

If Equation S1 and S2 are combined with the differential model equations in the text, the relationship between k_{max} and the physiochemical properties of biochars (EC and EEC) can be obtained and interpreted as follows:

$$k_{max} = A_0 \cdot [k_0 \cdot X_s \cdot f + \alpha \cdot f \cdot [biochar]_{\mathrm{red}} + \beta \cdot X \cdot \lambda \cdot (1-f)] \qquad \text{Equation S3}$$

Table S2. The fitted parameters of PCP biodegradation in the presence of different biochars.

Conditions	A_0	D_0	B_0	r	p
Control	2.2095	9.3110	17.8519	0.9874	3.0×10^{-5}
BC400	4.1817	7.5998	15.9271	0.9903	1.5×10^{-5}
BC500	5.8719	6.1312	14.1136	0.9947	1.8×10^{-5}
BC600	10.4331	5.4201	9.2540	0.9973	2.7×10^{-4}
BC700	11.6433	4.5025	7.7598	0.9967	1.4×10^{-3}
BC800	12.3634	3.4892	7.2529	0.9939	8.6×10^{-3}
BC900	17.9742	3.2103	3.5096	0.9971	1.6×10^{-3}

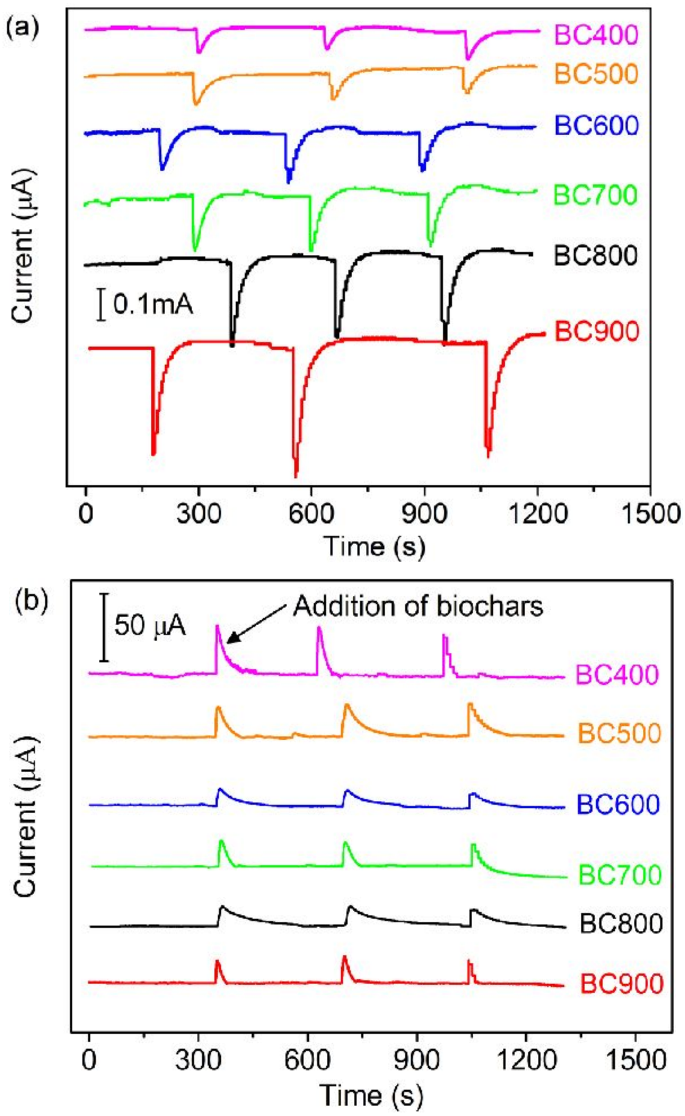

Fig. S1 Mediated electrochemical reduction (a, MER) and oxidization (b, MEO) of different biochars by the graphite electrode at applied potentials. Each peaks in this figure represented the responses of currents when a small amount of biochar suspensions (i.e., 1.0 mg of biochars) were spiked into the electrochemical cell. The peak areas were used to calculate the numbers of transferred electrons and EACs and EDCs of biochars.

Modification of biochar surface quinone groups. BC400 with the lowest EC was chemically modified by hydroquinone according to the method described by Perminova et al.[1]. Briefly, BC400 (1 g) was suspended in 50 mL of deionized water and its pH was adjusted to 7.0. Hydroquinone (0.5 g) was added to the suspension in the presence of a catalyzer (oxalic acid) and 1 g of 35% formaldehyde solution. The mixture was stirred and heated at 100 °C for 1 h. After cooling to room temperature, the product was centrifuged at 14,000 rpm for 15 min. The precipitate was washed with deionized water and centrifuged several times to avoid substrate sorption or residual. The modified biochar product (MBC400) was then freeze-dried and stored in a desiccator. Meanwhile, to introduce a model quinone compound to BC400 particles by sorption, BC400 (5 g) was equilibrated with 2.0 mmols of anthraquinone-2,6-disulfonate (AQDS) in deionized water for 72 h. Then the mixture was filtrated and the precipitate (denoted as BC400-AQDS) was air-dried for 24 h.

Surface quinone groups of BC900 (with the highest EAC) were destroyed according to the method reported previously by Zhang et al.[2]. Specifically, biochar (1 g) with the highest EAC (BC900) was selected and pretreated with 100 mL of 30% H_2O_2 solution for 48 h under stirring. Since biochar is a microporous material with large surface area, we prolonged the oxidation time from 15h to 48h. This will ensure that the oxidation reaction is thorough and decreases the content of quinone moieties of biochars as much as possible. The solution was then heated at 90 °C for 2 h and centrifuged at 14,000 rpm for 15 min. The precipitate (designated as MBC900) was washed several times with deionized water and freeze-dried. The biochar surface groups were characterized using Fourier-transform infrared spectroscopy (FTIR, Vector 33, Bruker Ltd., Germany).

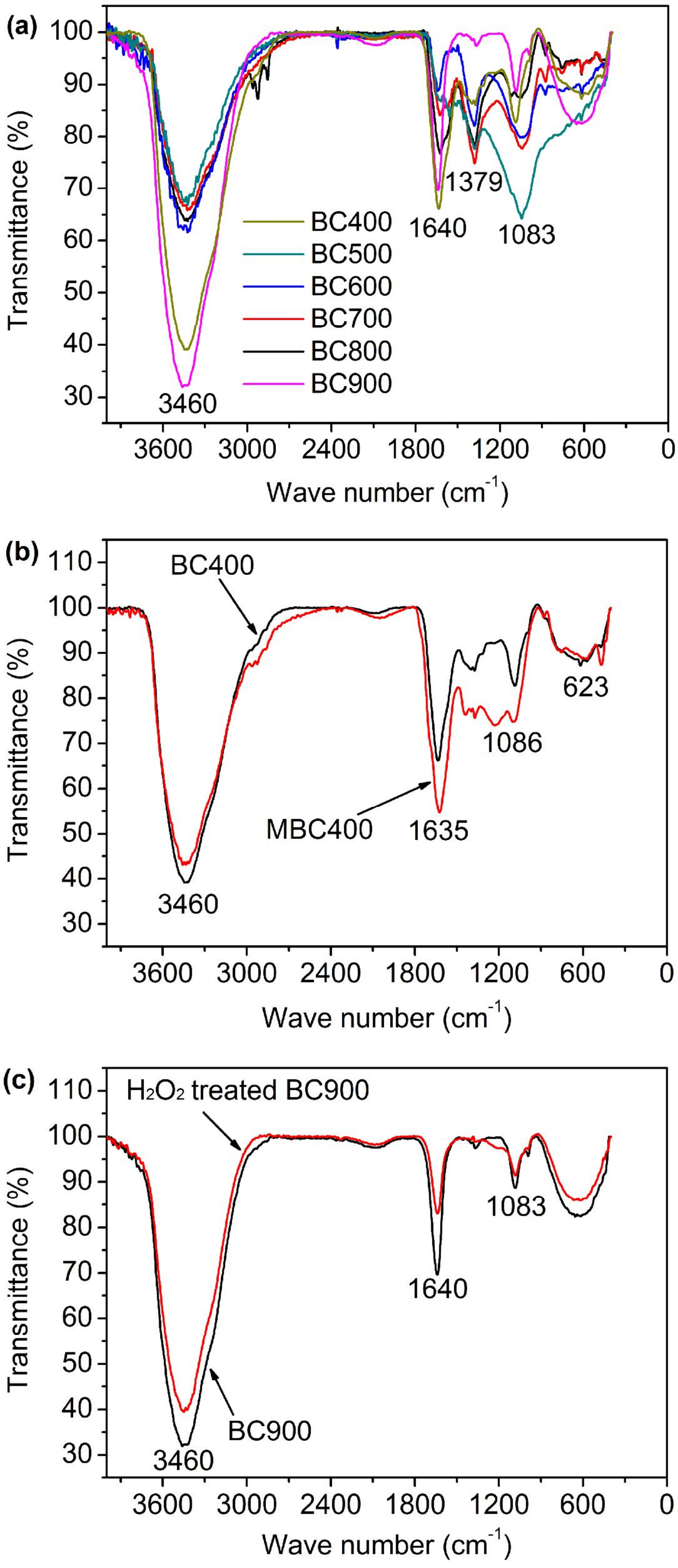

Fig. S2 Fourier Transform Infrared Spectrum of (a) six different biochars, (b) BC400 and BC400 modified by hydroquinone (MBC400), (c) BC900 and H_2O_2-treated BC900 (MBC900).

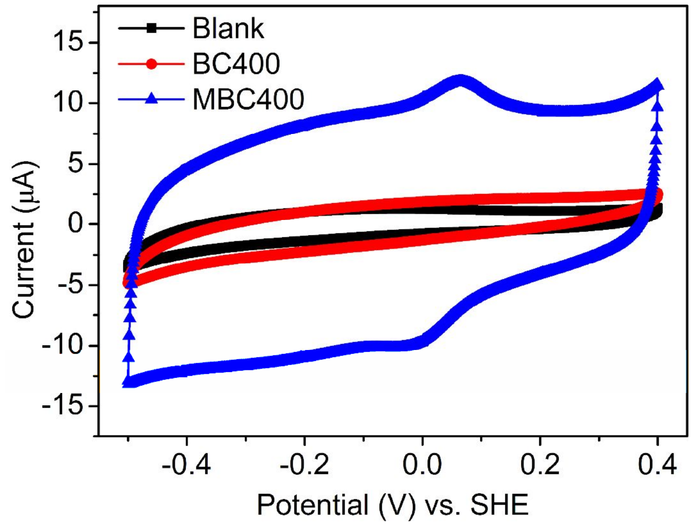

Fig. S3 Cyclic voltammetry (CV) characteristics of BC400 and BC400 modified by hydroquinone (MBC400) in the phosphate buffer solution (0.1M, 0.1M KCl, pH=7.0). The scan rate was 50 mV/s.

Procedures for extraction and quantification of PCP and its intermediate products. The adsorbed PCP in the biochar suspensions was extracted with ethanol (1:1, vol:vol) and the mixture was vibrated for 24 h in a vibrator at a rate of 200 rpm[3-5]. Then the samples were filtrated with a 0.22-μm filter membrane to remove biochar particles and the PCP concentrations in the filtrate were determined by high-pressure liquid chromatography at a detection wavelength of 295 nm (HPLC, Waters Alliance 1525-2487 system with a symmetry C_{18} column (5 μm, 4.6×250 mm^2, Waters, USA)), as previously described[3]. The mobile phase of HPLC was 1% (v/v) acetic acid in the methanol-water mixture (80:20, v/v) and the flow rate was 1 ml/min. All the concentrations of PCP reported were corrected from the dilution.

For GC/MS analysis, the intermediate products of PCP were acetylated with acetic anhydride in the presence of 0.1 M K_2CO_3 (pH=10.5) [3]. Then the reaction mixtures were extracted with hexane for three times and dehydrated by anhydrous sodium sulfate. Afterward, the intermediate products in hexane were concentrated by rotary evaporation and nitrogen gas to constant volume of 1ml. 1μl of aliquot was injected to gas chromatography-mass spectrometry (GC-MS, Thermo Trace-DSQ-2000 with electron ionization and an Agilent capillary column (0.25 mm × 30 m × 0.25 μm)). The protocols used for the determination of PCP and its intermediate products were as previously described by Tong et al.[3].

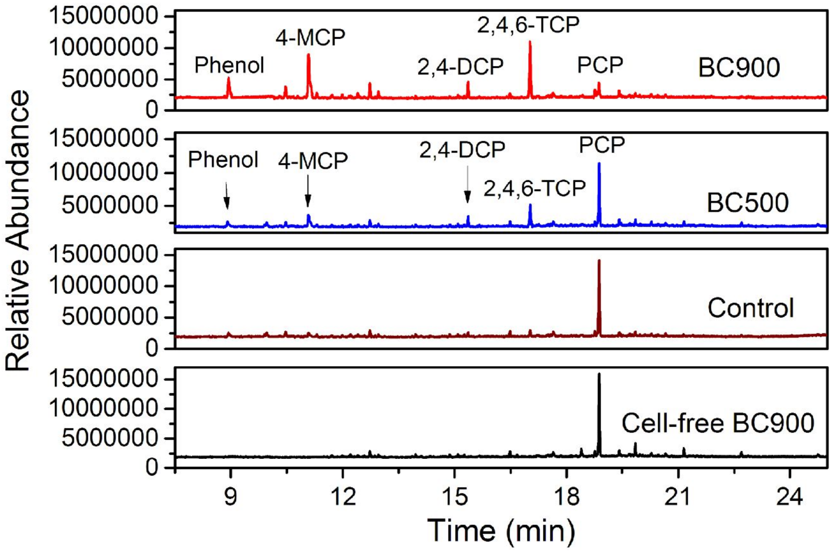

Fig. S4 GC/MS chromatographs of PCP intermediate products produced at 15 d under different biochar conditions with *G. sulfurreducens* (BC900, BC500 and Control) or without cells (cell-free BC900).

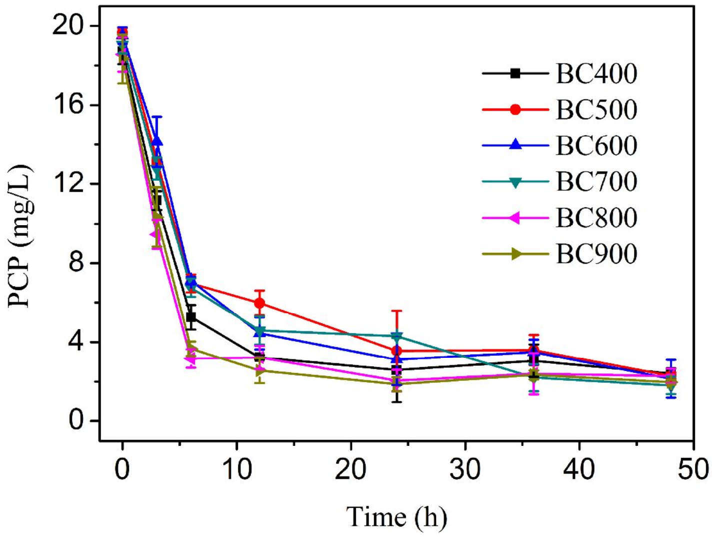

Fig. S5 Adsorption of PCP by different biochars. For this experiment, the concentrations of PCP in the aqueous phase (without extraction of the adsorbed PCP) in the presence of different biochars (2 g/L) were quantified by HPLC.

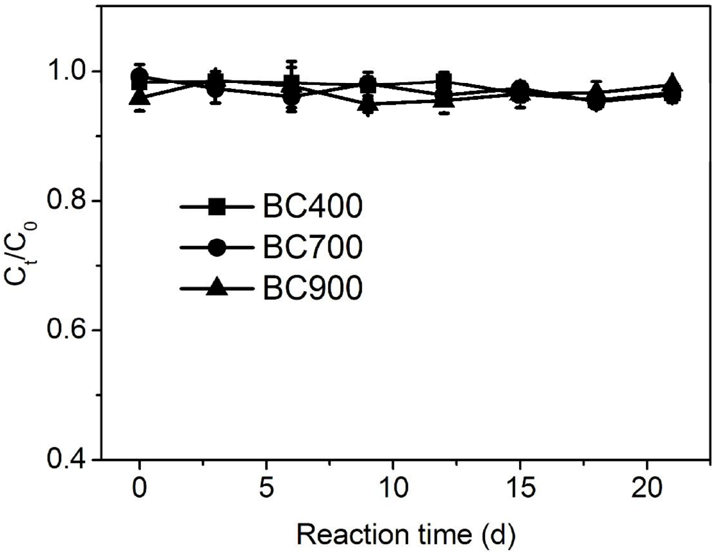

Fig. S6 Chemical transformation of PCP by different biochars in the cell-free NBAF culture mediums. PCP (20 mg/L) was incubated anaerobically with different biochars (2g/L) at 30 °C for 21 d. At different times, the adsorbed PCP in the samples were extracted with ethanol and filtrated to remove biochars, followed by quantification using HPLC. Error bars represent ± SD (n= 3). Since no PCP intermediate product was detected by GC/MS, the disappearance of PCP (~3%) was probably due to the slight extraction loss caused adsorption by biochars. Based on these data, the average PCP extraction efficiency at different times was 97.5 ± 1.26%, 97.02 ± 1.73% and 96.78 ± 1.96% for BC400, BC700 and BC900, respectively (n=24).

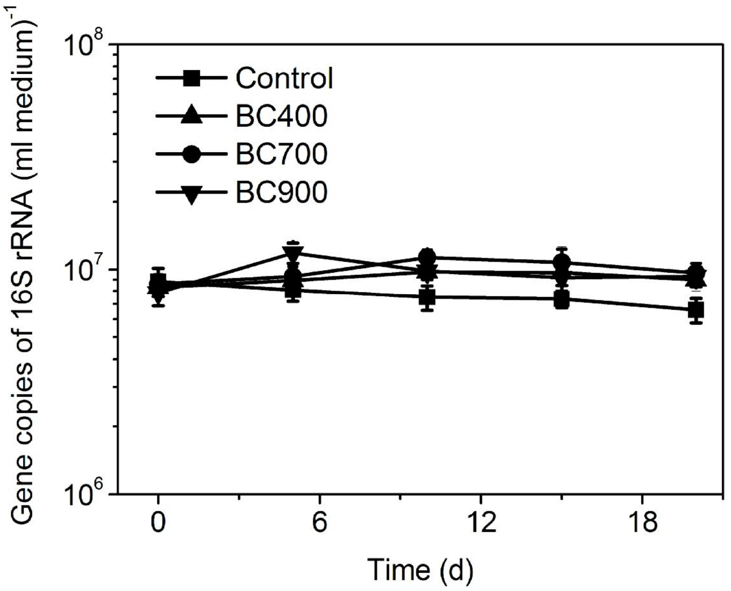

Fig. S7 Time courses of the biomass of *G. sulfurreducens* during PCP biodegradation in the absence or presence of biochars. For this experiment, real-time fluorescent quantitative PCR (qPCR) of the 16S rRNA gene numbers of *G. sulfurreducens* was performed to determine the biomasses as previously described[3,6].

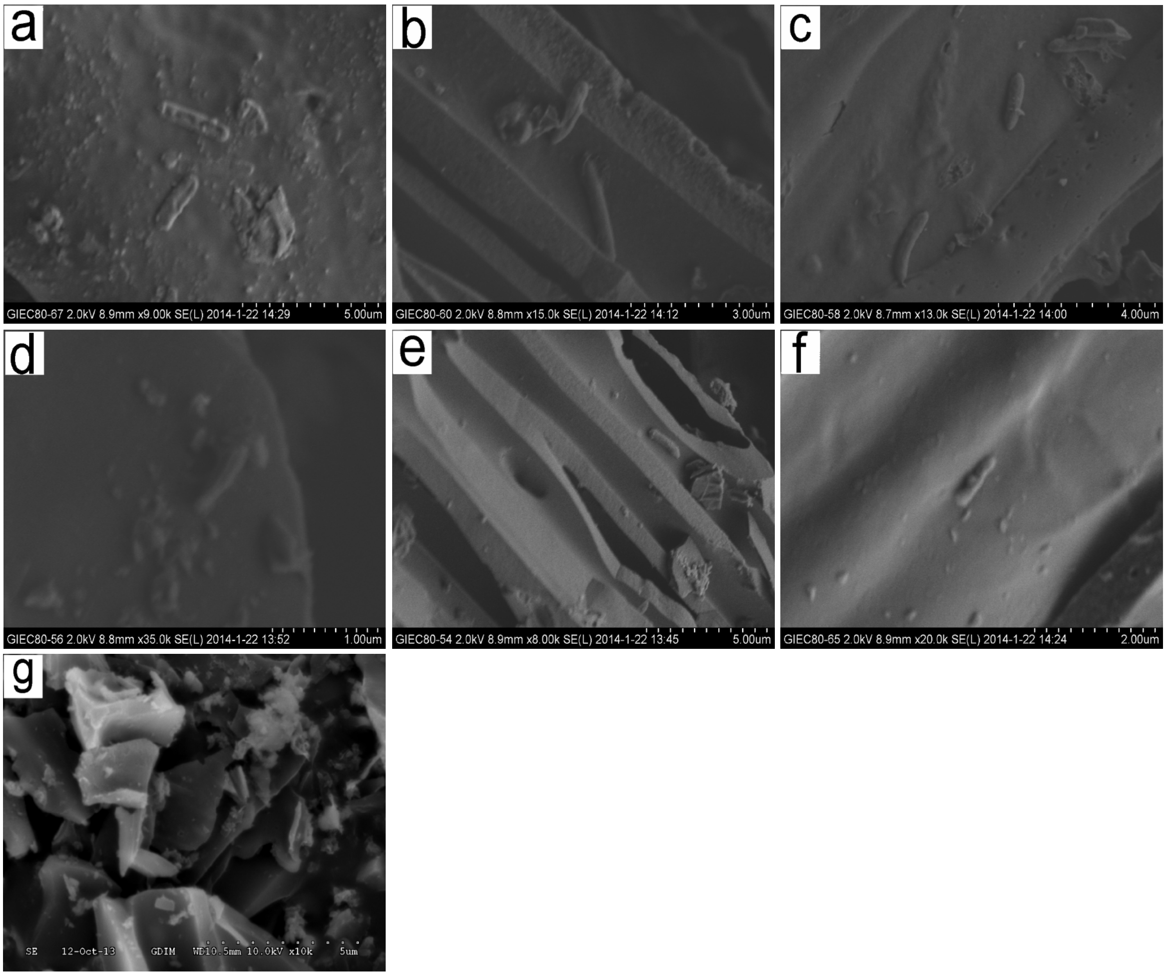

Fig. S8 SEM images of *Geobacter sulfurreducens* growing on the surface of biochars during the reductive biodegradation of PCP. At the end of PCP degradation kinetic experiments (21 d), biochars were harvested from the NBAF mediums by filtration at 0.45 μm and then cells on the biochar partilces were fixed with a 2.5% glutaraldehyde solution for 6 h, followed by a gradual dehydration using 25%, 50%, 75%, and 100% ethanol solutions, respectively. All the samples were freeze-dried and spray-coated with a thin film of platinum for SEM observation at 20kV. (a) BC400, (b) BC500, (c) BC600, (d) BC700, (e) BC800, (f) BC900, (g) sterile BC900.

Reference

1. Perminova, I. V. *et al.* Design of quinonoid-enriched humic materials with enhanced redox properties. *Environ. Sci. Technol.* **39**, 8518–8524 (2005).

2. Zhang, C. F. & Katayama, A. Humin as an electron mediator for microbial reductive dehalogenation. *Environ. Sci. Technol.* **46**, 6575–6583 (2012).

3. Tong, H., Hu, M., Li, F. B., Liu, C. S. & Chen, M. J. Biochar enhances the microbial and chemical transformation of pentachlorophenol in paddy soil. *Soil Biol. Biochem.* **70**, 142–150 (2014).

4. Bryant, F. O., Hale, D. D. & Rogers, J. E. Regiospecific dechlorination of pentachlorophenol by dichlorophenol-adapted microorganisms in freshwater, anaerobic sediment slurries. *Appl. Environ. Microbiol.* **57**, 2293–2301 (1991).

5. Khodadoust, A. P., Suidan, M. T., Sorial, G. A. & Dionysiou, D. D. Desorption of pentachlorophenol from soils using mixed solvents. *Environ. Sci. Technol.* **33**, 4483–4491 (1999).

6. Xu, J. L., Zhuang, L., Yang, G. Q., Yuan, Y. & Zhou, S. G. Extracellular quinones affecting methane production and methanogenic community in paddy soil. *Microb. Ecol.* **66**, 950–960 (2013).
